# Extracellular Vesicles as Enabling Biomarkers for New Approach Methodologies to Support the US Food and Drug Administration Modernization Act 3.0

**DOI:** 10.1002/jev2.70313

**Published:** 2026-05-28

**Authors:** Andrew Rowland, Carl Kirkpatrick, A David Rodrigues, Uli Frevert, Franziska Haderk, David W Greening, Andrew J McLachlan, Ashley Hopkins, Fabrice Lucien, Rienk Nieuwland

**Affiliations:** ^1^ College of Medicine and Public Health Flinders University Adelaide Australia; ^2^ Monash Institute of Pharmaceutical Sciences Monash University Melbourne Australia; ^3^ DMPK, Translational Medicine Incyte Wilmington Delaware USA; ^4^ Translational Medicine Madrigal Pharmaceuticals Conshohocken Pennsylvania USA; ^5^ Global ExpMed Oncology Boehringer Ingelheim Biberach Germany; ^6^ Baker Heart and Diabetes Institute Melbourne Australia; ^7^ Baker Department of Cardiovascular Research Translation and Implementation La Trobe University Melbourne Australia; ^8^ Baker Department of Cardiometabolic Health University of Melbourne Melbourne Australia; ^9^ Sydney Pharmacy School University of Sydney Sydney Australia; ^10^ Department of Urology, Mayo Clinic Rochester Minnesota USA; ^11^ Department of Immunology, Mayo Clinic Rochester Minnesota USA; ^12^ Department of Laboratory Medicine Amsterdam UMC Amsterdam Netherlands

## Abstract

The FDA Modernization Act 3.0 represents a pivotal shift in biomedical research by formally removing the requirement for animal testing and enabling regulatory acceptance of New Approach Methodologies (NAMs). This transition creates an urgent need for robust, human‐relevant biomarkers that can anchor NAM‐generated data within regulatory decision‐making, particularly for first‐in‐human studies. Extracellular vesicles (EVs), owing to their biological stability, accessibility and rich molecular cargo, may be well positioned to fulfil this role. EVs reflect dynamic cellular states and intercellular communication, making them mechanistically informative readouts of pharmacology, toxicity, immune modulation and disease biology across human‐derived model systems.

This Perspective outlines how EV‐based biomarkers can support the qualification, standardization, and regulatory acceptance of NAMs, with particular emphasis on *ex vivo* platforms such as organoids, microtissue systems, organ‐on‐chip devices and perfused tissue slices. We highlight the advantages of EVs for longitudinal, non‐destructive sampling, representation of human variability, and integration with quantitative systems pharmacology and physiologically based pharmacokinetic models. Finally, we discuss the scientific, operational and regulatory challenges that must be addressed and argue that coordinated leadership from the International Society for Extracellular Vesicles (ISEV) is essential. The convergence of regulatory reform and EV science presents a timely call to action to establish EVs as foundational biomarkers in next‐generation, human‐centric drug development.

**Abbreviations**
EV: Extracellular vesicle
FDA: US Food and Drug Administration
ISEV: International Society for Extracellular Vesicles
MISEV: Minimal Information for Studies of Extracellular Vesicles
NAM: New Approach Methodology
PBPK: Physiologically based pharmacokinetic
PET: Positron emission tomography
QSP: Quantitative systems pharmacology

## 1 Introduction

The US Food and Drug Administration (FDA) Modernization Act 3.0 builds on Act 2.0 and represents a watershed moment in biomedical research and drug development. By explicitly removing the statutory requirement for animal testing and accepting results from New Approach Methodologies (NAMs) as evidence for regulatory submissions, the Act promotes a shift toward more effective, human‐relevant science (Han 2023, FDA 2025c, FDA 2025a). Complemented by the FDA's roadmap to reduce animal use (FDA 2025c), these regulatory updates elevate *in vitro* systems, organoids, microphysiological devices, *in chemico* methods and *in silico* computational modelling, collectively termed NAMs, from experimental adjuncts to central pillars of nonclinical evaluation for investigational therapeutic products.

The FDA roadmap emphasizes that this transition will be supported by a diverse ecosystem of platforms, rather than any single replacement technology. Alongside complex *in vitro* and *in silico* models, the FDA highlights the growing role of *ex vivo* human models such as organ slices. The roadmap also acknowledges the potential of human microdosing studies combined with advanced imaging, such as positron emission tomography, to generate early pharmacokinetic and biodistribution data directly in humans.

For drug developers, this legislative and regulatory pivot will reshape how nonclinical and early clinical evidence packages are designed, sequenced and justified. Key pharmacology and safety questions, particularly for modalities like complex biologics which are poorly recapitulated in animals, will increasingly be answered by human‐relevant models rather than traditional animal studies. Within this setting, human‐derived biomarkers that can bridge NAM readouts into first‐in‐human studies are likely to become central elements of clinical development programmes and regulatory evidence rather than optional additions, especially if easily implemented with low burden and at scale.

**FIGURE 1:**
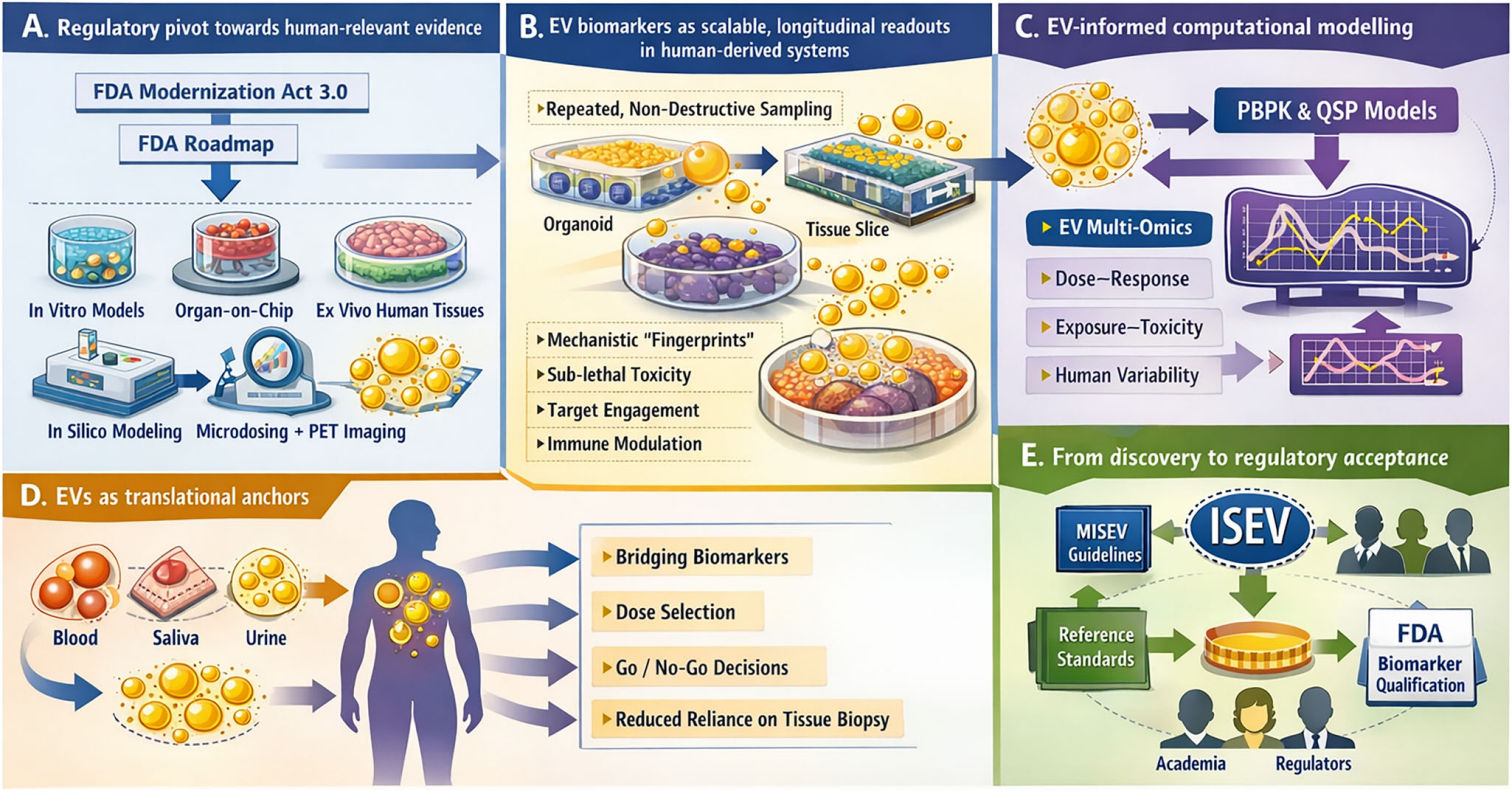
**Extracellular vesicles (EVs) as integrative, human‐relevant biomarkers supporting New Approach Methodologies (NAMs) under FDA Modernization Act 3.0**.EVs connect human‐derived NAM platforms, computational modelling and early clinical studies within emerging regulatory frameworks. (A): Regulatory reforms enable NAM‐based evidence to replace routine animal testing. (B): EVs provide scalable, non‐destructive and longitudinal readouts in human‐derived systems. (C): EV data inform physiologically based pharmacokinetic (PBPK) and quantitative systems pharmacology (QSP) models. (D): Biofluid EVs translate NAM signatures to first‐in‐human studies. (E): Standardization and qualification enable regulatory adoption of EV biomarkers.

Across this expanding landscape of NAMs, a fundamental challenge emerges: the need for biomarkers that faithfully reflect human biology, are mechanistically grounded and meet regulatory standards for qualification and reproducibility, and clearly defined in contexts of use. Extracellular vesicles (EVs), especially tissue‐specific EVs isolated from plasma, saliva and excreta, given their stability, accessibility and molecular richness (Théry et al. 2018, Welsh et al. 2024, Newman and Rowland 2025, Rai et al. 2025), represent a promising, but still maturing approach. EVs represent one of several emerging human‐relevant biomarker modalities under consideration for NAM integration. Soluble protein biomarkers, multi‐omics (e.g., transcriptomic, metabolomic, proteomic or lipidomic) signatures, cell‐free nucleic acids, imaging approaches, and functional microphysiological readouts may, in some contexts, offer more direct or more scalable solutions. EV‐based biomarkers will need to demonstrate added value, whether through improved sensitivity, risk prediction and monitoring, mechanistic specificity, or translational continuity, relative to these established approaches. This Perspective outlines how EV‐based biomarkers may support the implementation, standardization, and regulatory acceptance of NAMs, the importance of reproducibility and harmonization, and why this moment constitutes a call to action for the EV research community, particularly under the leadership of the *International Society for Extracellular Vesicles* (ISEV) (**Figure** **1**).

## 2 Extracellular Vesicles and the Evolving Demand for Human‐Relevant Biomarkers

NAMs generate extensive and multidimensional datasets (Herland et al. 2020, Hall et al. 2020, Ingber 2022). To interpret such data, regulators require validated biomarkers that map onto known human pathophysiology and are reproducible across laboratories. EVs have attributes that may position them to contribute to this role, provided key technical and validation hurdles are addressed. The protein, lipid, nucleic acid and metabolite cargo contained in EVs reflects dynamic changes in cellular state (Newman et al. 2020, Newman et al. 2022). This characteristic enables EVs to serve as readily implementable and sensitive readouts of disease state, between subject variability, toxicity, immune modulation, therapeutic engagement and tissue microenvironmental processes with potential relevance to go/no‐go and dose selection decisions within development programmes. For example, in complex multicellular organ‐on‐chip systems where multiple cell types contribute to toxicity or immune modulation, EV profiling may capture coordinated pathway perturbations that are not reflected by single soluble cytokine measurements (Ingber 2022, Nguyen et al. 2022, Zhang et al. 2025). While EVs have already gained traction as diagnostic and theragnostic tools in diverse clinical settings (Rowland et al. 2019, Useckaite et al. 2021, Yáñez‐Mó et al. 2015, Lane et al. 2018, Greening et al. 2025), their integration into NAMs could provide the mechanistic and translational biomarkers needed to anchor NAM data within the regulatory process involving first‐in‐human clinical trials.

It is important to distinguish scenarios in which EV‐based biomarkers may offer advantages over simpler or more established readouts. Soluble protein biomarkers and targeted transcriptomic assays can provide sensitive and scalable measurements of defined pathways and may be preferable when a single mechanism is under investigation. A notable example is the fidelity gain achieved from the analysis of miRNA markers in tissue‐specific EVs isolated from blood. Newman, Useckaite et al. (Newman et al. 2022) demonstrated that quantification of disease‐associated miRNAs in liver‐specific EVs, but not the total EV population or whole blood, enabled discrimination between healthy controls, mild and severe liver disease. Likewise, imaging‐based readouts in organoids, microtissue platforms and organ‐on‐chip systems provide spatial and functional information that EV analysis cannot replace. EV‐based biomarkers may add value in contexts where multiplexed, multi‐omics information reflecting integrated cellular state and intercellular communication is required, particularly when longitudinal, non‐destructive sampling is advantageous. However, such added complexity must translate into demonstrable incremental decision value to justify implementation.

## 3 Extracellular Vesicles as *Ex Vivo* Biomarkers in Next‐Generation Human Models

The interface between EV biology and advanced human‐derived systems represents promising opportunities for innovation. Organoids, organ‐on‐chip platforms, perfused tissue slices and 3D co‐cultures recapitulate essential aspects of human physiology (Lancaster and Knoblich 2014, Bhatia and Ingber 2014, van Midwoud et al. 2010), but they all require validated, scalable and interpretable biomarkers to translate their biological sophistication into regulatory utility. Well‐designed and executed EV protocols can provide such a readout to support qualification of the NAMs.

EVs released into defined culture media or organ‐on‐chip sampling chambers can be repeatedly collected and, in some cases, stepwise production (Lenoir et al. 2026) and continuous sampling from enclosed microfluidic systems (Hassanzadeh‐Barforoushi et al. 2025) for analysis, with multi‐omics profiles that reflect tissue‐specific disease states, stress responses, adaptation, and intercellular communication (Blaser et al. 2023). Emerging studies already demonstrate the feasibility and value of EVs as functional readouts in advanced human‐derived model systems. EVs harvested from organoids and microphysiological systems capture cell‐type‐specific and state‐dependent molecular information, including pathway activation, stress responses and disease‐relevant signalling, supporting their use as non‐destructive biomarkers within complex *in vitro* platforms (Nguyen et al. 2022, Zhang et al. 2025). Mechanistic EV “fingerprints” can be developed for sub‐lethal toxicity, immune activation or suppression, tumour–stroma signalling and pathway modulation. These so‐called “signatures” not only complement functional metrics within NAM systems but also improve causal inference between molecular perturbations and phenotypic outcomes (Abdal Dayem et al. 2025).

A significant advantage of EV biomarkers in *ex vivo* systems is the opportunity to collect serial longitudinal samples (Rodrigues and Rowland 2019). The capacity for longitudinal (repeat) sampling is particularly useful and important for *in vivo* studies, including human studies. Currently, spatially confined tissue biopsies are typical examples of endpoints that can be collected and analysed once or twice in a clinical study, which is a major hurdle for disease phenotyping and drug development. Unlike tissue biopsy, EV biomarkers may capture aspects of molecular heterogeneity not accessible through spatially confined biopsies, though the extent of incremental sensitivity remains to be systematically defined. If realised, this creates the potential to capture the complete molecular and phenotypical heterogeneity of a patient's disease, thereby providing a more accurate snapshot of toxicity signals, drug sensitivity/resistance and correlative markers of functional endpoints. Time‐resolved EV analyses can identify early toxicity signals, define dose–response kinetics, assess reversibility following drug washout and correlate molecular signatures with real‐time functional endpoints (e.g., barrier integrity, electrophysiology). Such dynamic EV fingerprint measurements may provide earlier or more sensitive molecular indicators of tissue perturbation in human‐derived systems, although systematic head‐to‐head benchmarking against established markers remains limited. These capabilities may complement, and in some contexts may offer advantages in specific contexts, particularly where animal models have limited human translatability, although comparative performance data remain sparse. These propositions remain, in part, forward‐looking hypotheses that will require prospective benchmarking against established soluble biomarkers, pre‐analytical variance and standardized analytical assessments, imaging endpoints, and animal‐based readouts.

Moreover, EVs offer a path for representing human variability within NAMs. EV cargo from patient‐derived organoids or co‐cultures inherently reflects donor‐specific biology, enabling stratified analyses that incorporate genetic and environmental diversity. When similar EV signatures are detected in patient biofluids, they can serve as translational anchors that strengthen confidence in NAM‐derived predictions.

In addition, these longitudinal EV outputs also provide key building blocks for highly mechanistic (and biologically plausible) *in silico* Quantitative Systems Pharmacology (QSP) models. The utility of such models is their ability to learn and confirm optimal dose, efficacy and toxicity in the whole human‐specific system. For example, in a liver‐chip model, EV‐derived hepatocyte injury markers (e.g., liver‐enriched miRNAs or injury‐associated protein cargo(Newman et al. 2022, Newman et al. 2022)) could be incorporated as dynamic indicators of hepatocyte stress or loss, informing model parameters governing cell viability, injury kinetics or recovery rates within QSP frameworks. Similarly, EV‐associated cytokines or immune‐regulatory proteins released from tumour–immune organ‐on‐chip systems could be used to parameterize intercellular signalling strengths or feedback loops that modulate immune activation and tumour response dynamics. Similarly, in a physiologically based pharmacokinetic (PBPK) context, and consistent with prior demonstrations that plasma‐derived small EVs reflect hepatic enzyme and transporter expression (Achour et al. 2021, Rodrigues et al. 2021), liver‐specific EV‐derived protein or mRNA signatures quantified in liver‐chip systems or other hepatic NAM platforms may serve as dynamic, non‐destructive indicators of hepatic enzyme and transporter abundance under defined exposure conditions. Such EV‐derived measurements could inform estimates of intrinsic clearance enzyme induction parameters, or transporter activity terms within PBPK frameworks, thereby strengthening the biological basis for extrapolating metabolism and drug–drug interaction risk from hepatic NAM platforms to clinical settings. **Industrialization of Extracellular Vesicle Biomarkers for New Approach Methodology‐Enabled Drug Development**

For EV biomarkers to realise their potential in the NAM era, EVs will need to be considered not only at the level of individual experiments but also across the full trajectory of drug discovery and development. From the perspective of drug development programmes and regulatory evaluation, this may, in selected therapeutic areas, justify explicit EV biomarker strategies for specific therapeutic areas that span discovery, nonclinical safety, early clinical pharmacology and, where justified, late‐phase and post‐marketing studies. Delivering such a strategy in practice will require assay platforms and workflows in sample collection, storage, and analytical processes that are controlled and sufficiently robust for routine use. High‐throughput EV isolation and characterization must be scalable to the hundreds or thousands of samples generated in multi‐centre clinical trials. This is particularly important to validate EV‐based markers not just against established preclinical models but also based on the predictive value for human experience, including previously developed physiologically based pharmacokinetic—quantitative systems pharmacology (PBPK‐QSP) models from early *in‐vitro*, organoid, and perfused tissue slices. From the perspective of clinical development, EV‐based liquid biopsies also offer practical and ethical advantages. Because EVs can be measured repeatedly from peripheral blood or other minimally invasive matrices, they have the potential to reduce reliance on serial tissue biopsies that are burdensome for patients, costly, logistically and ethically challenging and ultimately can be a barrier for recruitment and retention of participants in clinical trials.

## 4 Regulatory Pathways and Role for the International Society of Extracellular Vesicles

As EV science moves closer towards regulatory application, structured pathways for engagement are essential. The FDA has already identified EVs as a regulatory science priority through its work on EV‐based therapeutics and biomanufacturing (Wang et al. 2024), establishing a conceptual precedent for EV‐based biomarkers in NAM contexts. Early contexts of use might include EV‐derived safety biomarkers (such as early markers of hepatocyte injury in liver‐chip models), pharmacodynamic biomarkers reporting target engagement in tumour‐on‐chip systems or bridging biomarkers linking *ex vivo*, *in vivo* (where still used) and early clinical data.

Progressing these biomarkers through the FDA Biomarker Qualification Program will require communities capable of harmonizing methods, generating shared datasets and uniting expertise across academia, industry and regulatory bodies. Here, the International Society for Extracellular Vesicles (ISEV) should take a leading role. As the leading global scientific society strongly promoting EV standardization and reproducibility, ISEV is uniquely positioned to coordinate EV–NAM integration. ISEVs history of generating consensus guidelines, such as MISEV (Théry et al. 2018, Welsh et al. 2024), establishes the foundational infrastructure needed for reproducibility, transparency and methodological convergence. An ISEV‐led initiative could define performance expectations for EV‐based NAM biomarkers, establish reference materials and support training programs tailored specifically to *ex vivo* and microphysiological systems.

For sponsors, a risk‐balanced approach to regulatory engagement is likely to be most tractable. In many programmes, EV biomarkers will enter as exploratory endpoints in early‐phase trials or as internal decision‐making tools in NAM‐based screening. As such, they will provide important information in early‐stage drug development. A progressive elevation to secondary or, in selected cases, primary endpoints may be limited and is, in general, only possible once assay performance and clinical relevance have been demonstrated. This stepwise maturation mirrors experience with other safety and pharmacodynamic biomarkers qualified through the FDA Biomarker Qualification Program (FDA 2025b) and analogous initiatives in Europe and elsewhere. Likewise, regulatory adoption of EV‐based biomarkers is likely to be incremental and context‐specific. Agencies appropriately require robust evidence of analytical validity, clinical relevance, and defined contexts of use before accepting novel biomarkers in decision‐making frameworks. EV‐based readouts in NAM systems will therefore initially serve as supportive or exploratory evidence streams rather than replacements for established endpoints. Premature positioning of EVs as universal translational bridges risks undermining confidence in the field. A measured, data‐driven progression toward qualification will be essential.

## 5 Scientific and Practical Challenges

Realizing the potential of EV biomarkers in NAMs requires addressing several scientific and operational challenges. Pre‐analytical and analytical variables, including media composition, serum supplementation, culture architecture, and isolation techniques, all strongly influence EV yield, presence of confounders, and content (Welsh et al. 2024). Standardization, reference materials and rigorous and transparent reporting are essential to reduce technical variability (Lucien et al. 2023). High‐throughput workflows will require miniaturized, automated EV isolation and analysis technologies compatible with small‐volume NAM systems. Integrating high‐dimensional EV‐omics datasets with functional outputs and *in silico* computational models, for example, PBPK and QSP, will be critical for producing mechanistically interpretable data suitable for regulatory review. EV biomarkers must be benchmarked against legacy endpoints to demonstrate equivalence or superiority, ideally by retrospectively analysing standardized sets of reference compounds with known toxicological liabilities and clinical outcomes, enabling direct comparison with established nonclinical decision frameworks. While not the ultimate goal of EVs in this setting, these foundational studies provide evidence of validity for future applications.

The intrinsic heterogeneity of EVs is a central scientific challenge. EV populations comprise a continuum of subtypes derived from multiple biogenetic pathways, with overlapping size distributions and partially shared molecular markers (Willms et al. 2018, Silva et al. 2025). Current isolation approaches do not fully resolve these subpopulations, and differences in enrichment strategy can substantially influence measured cargo profiles. As a result, the biological meaning of a given EV “signature” will depend strongly on methodological context (Silva et al. 2025). Without clear operational definitions and reference materials, this heterogeneity poses risks for reproducibility and regulatory interpretability.

Between‐laboratory variability remains a significant barrier. Even with adherence to MISEV guidelines (Welsh et al. 2024), differences in centrifugation protocols, chromatography matrices, antibody specificity, and downstream omics platforms can produce materially different outputs. For regulatory application, assay performance in terms of precision, accuracy, sensitivity, specificity, stability and between‐site reproducibility must be defined with the same rigour applied to established bioanalytical assays (Nieuwland et al. 2020, Nieuwland et al. 2022). At present, most EV biomarker assays remain research‐grade rather than regulatory‐grade, and broad deployment will require substantial method maturation. In particular, few EV‐based assays have undergone formal bioanalytical validation under Good Laboratory Practice (GLP) or demonstrated multi‐site reproducibility across independent laboratories. Standardized reference materials, assay performance benchmarks, and predefined acceptance criteria analogous to those required for qualified safety or pharmacodynamic biomarkers are not yet widely established. As such, substantial coordinated validation efforts will be required before EV biomarkers can routinely support regulatory decision‐making. Indeed, at present, no EV‐based biomarker has been fully qualified through the FDA Biomarker Qualification Program for use in drug development, underscoring the developmental distance between current research applications and regulatory‐grade implementation.

Practical feasibility must also be considered. High‐throughput, cost‐effective EV isolation compatible with small‐volume organ‐on‐chip systems and large‐scale clinical trials is not yet universally available. For many programmes, current EV multi‐omics profiling remains too slow and costly for routine deployment at clinical‐trial scale, necessitating targeted, fit‐for‐purpose panels as interim solutions. Automation, miniaturization and standardized reference materials will be essential to reduce operator‐dependent and pre‐analytical variability, to ensure that EV biomarker platforms are effective and economically sustainable within industrial drug development. Finally, caution is warranted in attributing mechanistic meaning to EV cargo changes. EV‐associated molecules may reflect passive release, cellular stress, or altered clearance rather than specific pathway modulation. Systematic benchmarking against orthogonal functional readouts and legacy endpoints will therefore be critical to avoid overinterpretation. These barriers can be addressed through fit‐for‐purpose assay tiering, multi‐site ring trials, and shared reference materials aligned to defined contexts of use. Ultimately, EV biomarkers must demonstrate not only analytical validity but also incremental predictive or decision‐making value relative to simpler and more scalable biomarker modalities within clearly defined contexts of use.

## 6 A **Call to Action for the Extracellular Vesicle Community**

The convergence of regulatory change, technological capability and scientific readiness creates an unprecedented opportunity for the EV field, but also a responsibility. The FDA Modernization Act 3.0 does not merely allow alternative methods; it compels the scientific community to develop human‐relevant tools that fulfil regulatory needs (Han 2023). EV biomarkers have the potential to contribute to this transformation, but only if the EV community acts collectively and decisively. Under the coordinated leadership of ISEV, EV researchers should establish focused EV–NAM working groups, develop open‐access EV datasets from well‐characterized *ex vivo* systems, incorporate biomarker qualification principles into study design and embed EV endpoints into ongoing NAM consortia (Figure **1**). As initial priorities, ISEV and the broader EV community could (i) define fit‐for‐purpose reference panels consisting of standardized biofluids and NAM‐derived samples with known perturbations; (ii) coordinate inter‐laboratory ring trials to quantify analytical variability across isolation and detection platforms, following an established ISEV model that has been applied in other settings (Bettin et al. 2025) (iii) establish minimum performance criteria for EV‐based assays intended for regulatory contexts, including predefined thresholds for precision, accuracy and inter‐site reproducibility; and (iv) prioritize a limited number of clearly defined “contexts of use,” such as early hepatotoxicity detection in liver‐chip systems or assessment of enzyme induction potential, to focus validation efforts. Concentrating on well‐scoped use cases may accelerate qualification relative to attempting broad, cross‐therapeutic deployment.

A fragmented approach, in which individual laboratories pursue isolated EV biomarker strategies without harmonized standards or shared datasets, would significantly slow regulatory acceptance. Without coordinated validation efforts, the field risks generating a proliferation of non‐comparable signatures that lack sufficient reproducibility for qualification. Collective action is therefore not merely advantageous but necessary to avoid dilution of scientific credibility. Likewise, collaboration with industry (pharma/biotech) and their engagement in guideline and protocol development are critical in this context. The involvement of these key stakeholders will minimise the significant risk of moving in the wrong direction. By doing so, the field can ensure that EVs become foundational evidence streams in future nonclinical development.

## 7 Conclusion

The FDA Modernization Act 3.0 presents a rare inflection point in the evolution of biomedical science. EVs possess multiple key attributes that may position them to serve as mechanistic, human‐centred biomarkers needed to realise the promise of NAMs, especially prior to first‐in‐human clinical studies. However, realising this potential will depend on rigorous standardization, careful validation, and realistic positioning within regulatory frameworks. In this regard, it is envisioned that EV‐based biomarkers can bridge preclinical NAM to Phase I clinical studies. With leadership from ISEV and unified action across the global EV community, *ex vivo* EV biomarkers may be able to add to scientific evidence in regulatory decision‐making, advancing not only the EV field, but also a more ethical, efficient and human‐relevant future for drug development and regulatory science.

## Data Availability

Data sharing not applicable to this article as no datasets were generated or analysed during the current study.
